# Supplementary material for: Association between serum Klotho and the prevalence of osteoarthritis: A cross-sectional study from NHANES 2007–2016
Source: PLoS One. 2024 Nov 18;19(11):e0312562. doi: 10.1371/journal.pone.0312562 (PMC11573205; doi:10.1371/journal.pone.0312562)
Supplement: S1 Table — (DOCX) [file pone.0312562.s001.docx]

**S1 Table.** Baseline characteristics of the Chinese population.

| Variables | Total  (N = 107) | Non-osteoarthritis  (N = 56) | Osteoarthritis  （N = 51） | P-value |
| --- | --- | --- | --- | --- |
| Sex (%) |  |  |  | 0.111 |
| Female | 65(60.75) | 30(53.57) | 35(68.63) |  |
| Male | 42(39.25) | 26(46.43) | 16(31.73) |  |
| Age (years) | 54.26(1.64) | 55.11(12.77) | 63.55(7.33) | < 0.001 |
| Race (%) |  |  |  | 0.194 |
| Han | 66(61.68) | 30(53.57) | 36(70.59) |  |
| Zhuang | 38(35.51) | 24(42.86) | 14(27.45) |  |
| Yao | 3(2.80) | 2(3.57) | 1(1.96) |  |
| Klotho (U/L) | 54.74(5.98) | 84.46(31.84) | 20.52(13.47) | < 0.001 |

Continuous variables were expressed as mean (standard error) and categorical variables were expressed as frequencies (percentages).
