# Supplementary material for: Association between serum Klotho and the prevalence of osteoarthritis: A cross-sectional study from NHANES 2007–2016
Source: PLoS One. 2024 Nov 18;19(11):e0312562. doi: 10.1371/journal.pone.0312562 (PMC11573205; doi:10.1371/journal.pone.0312562)
Supplement: S2 Table — (DOCX) [file pone.0312562.s002.docx]

**S2 Table**. Association between serum Klotho and osteoarthritis in the Chinese population.

| Exposures | Model1  [OR (95% CI) *P*-value] | Model2  [OR (95% CI) *P*-value] |
| --- | --- | --- |
| Klotho (continuous) | 0.79(0.69,0.90) <0.001 | 0.77(0.66,0.90) 0.001 |

Model 1 was not adjusted for any variables.

Model 2 was adjusted for gender, race, and age.
